# Comment on: ‘Disclosure of diagnosis by parents and caregivers to children infected with HIV in Hawassa, southern Ethiopia: a multicentre, cross-sectional study’

**DOI:** 10.1093/inthealth/ihae061

**Published:** 2024-10-07

**Authors:** John Patrick C Toledo

**Affiliations:** Department of Theology and Religious Education, De La Salle University, 2401 Taft Avenue, 1004 Manila, Philippines

Dear Editor,

I recently came across the article entitled ‘Disclosure of diagnosis by parents and caregivers to children infected with HIV in Hawassa, southern Ethiopia: a multicentre, cross-sectional study’. This study offers insights into the dynamics of disclosing an HIV diagnosis to children, a subject of great importance given the psychological, social and health implications of such disclosures.

‘It is important to provide support and counselling to the children when their HIV status is disclosed’.^1^ The findings point out the complexities and challenges that parents and caregivers face when informing children about their HIV status. In Hawassa, the process of disclosure is influenced by numerous factors, including cultural norms, the perceived maturity of the child and concerns about stigma and discrimination. The study points out that while many caregivers acknowledge the importance of disclosure for adherence to treatment and psychosocial well-being, they often delay this process due to fear of negative emotional reactions and social repercussions.

The research identifies several key barriers to disclosure, such as the lack of proper guidelines and support systems for caregivers, the caregivers’ own emotional readiness and the potential for the child to inadvertently disclose his/her status to others, leading to stigmatization. Despite these challenges, the study also points out that when disclosure is handled with sensitivity and appropriate support, it can significantly enhance the child's understanding of their condition, improve adherence to antiretroviral therapy and foster a supportive family environment.

In light of these findings, the study encourages the development of culturally sensitive, evidence-based regulations and support programs to assist caregivers in the disclosure process. Training healthcare providers to offer counselling and support can also play a crucial role in facilitating timely and effective disclosure.

‘Attitudes toward HIV-positive individuals are also stigmatized, as HIV infection is often equated to sin and immorality’.^2^ Reflecting on the situation in the Philippines, similar challenges are observed. The stigma surrounding HIV/AIDS remains a significant barrier to disclosure, and many parents and caregivers struggle with the decision of when and how to inform their children about their diagnosis. Cultural factors and societal attitudes towards HIV/AIDS contribute to the reluctance to disclose, fearing discrimination and social isolation.

Figure 1 depicts estimated annual new HIV infections among individuals ≥15 y of age from 2000 to 2021, showing both global trends and specific figures for the Philippines, emphasizing ongoing challenges in managing the epidemic.^3^ A holistic support system is essential for HIV patients, as it not only enhances their access to timely treatment and adherence, but also fosters a positive environment for psychological and emotional well-being, which is essential for effectively managing their health. The Philippines, like Ethiopia, faces the need for robust support systems and guidelines to assist caregivers in this delicate task. Programs aimed at reducing stigma and increasing public awareness about HIV/AIDS are crucial. Additionally, integrating counselling services within the healthcare system can provide much-needed support for families navigating the disclosure process.

**Figure 1.**
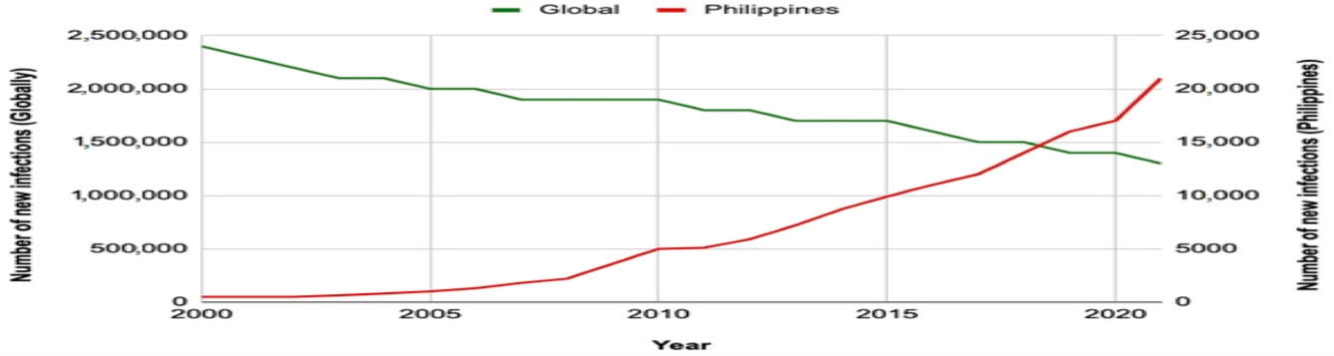
Estimated annual new HIV infections among individuals ≥15 y of age from 2000 to 2021 globally and in the Philippines, based on UNAIDS estimates. *Source*: Gangcuangco LMA, Eustaquio PC. The state of the HIV epidemic in the Philippines: progress and challenges in 2023. Trop Med Infect Dis 2023;8(5):258.

In both countries, community-based approaches and the involvement of various stakeholders, including healthcare providers, community leaders and support groups, can enhance the effectiveness of disclosure strategies. These initiatives should focus on educating the public about HIV/AIDS, promoting acceptance and creating a supportive environment for affected families.

The parallels between Ethiopia and the Philippines highlight the universal challenges of HIV disclosure to children and the need for comprehensive, culturally appropriate strategies to address these issues. The insights from the study in Hawassa can serve as a valuable reference for developing similar approaches in the Philippines and other regions facing similar challenges.

In conclusion, the article by Tari et al.^1^ provides essential evidence on the importance of sensitive and supported disclosure processes. By implementing culturally tailored guidelines and support systems, both Ethiopia and the Philippines can improve the well-being of children living with HIV and their families, fostering a more inclusive and understanding society.

## Data Availability

No new data were generated in support of this research.
